# Strategic Adaptation to Task Characteristics, Incentives, and Individual Differences in Dual-Tasking

**DOI:** 10.1371/journal.pone.0130009

**Published:** 2015-07-10

**Authors:** Christian P. Janssen, Duncan P. Brumby

**Affiliations:** 1 UCL Interaction Centre, University College London, London, United Kingdom; 2 Department of Experimental Psychology, Helmholtz Institute, Utrecht University, Utrecht, The Netherlands; University of California, San Francisco, UNITED STATES

## Abstract

We investigate how good people are at multitasking by comparing behavior to a prediction of the optimal strategy for dividing attention between two concurrent tasks. In our experiment, 24 participants had to interleave entering digits on a keyboard with controlling a randomly moving cursor with a joystick. The difficulty of the tracking task was systematically varied as a within-subjects factor. Participants were also exposed to different explicit reward functions that varied the relative importance of the tracking task relative to the typing task (between-subjects). Results demonstrate that these changes in task characteristics and monetary incentives, together with individual differences in typing ability, influenced how participants choose to interleave tasks. This change in strategy then affected their performance on each task. A computational cognitive model was used to predict performance for a wide set of alternative strategies for how participants might have possibly interleaved tasks. This allowed for predictions of optimal performance to be derived, given the constraints placed on performance by the task and cognition. A comparison of human behavior with the predicted optimal strategy shows that participants behaved near optimally. Our findings have implications for the design and evaluation of technology for multitasking situations, as consideration should be given to the characteristics of the task, but also to how different users might use technology depending on their individual characteristics and their priorities.

## Introduction

People choose to multitask in many daily settings, as illustrated in a recent special issue on multitasking [1]. For example, office workers frequently self-interrupt themselves throughout a typical day [2,3], switching activities every two to three minutes [4]. This desire to switch between activities remains even when performing activities that really should demand our complete and undivided attention. A topical example of this is driver distraction and the numerous reports of drivers using their phones to write and receive messages while driving (e.g., [5–7]).

A core question for multitasking research has been to consider whether people are good at multitasking (e.g., [8–11]). If people are not good at multitasking then maybe this behavior should be discouraged. At one level the answer to this question seems clear cut as there is an abundance of research demonstrating dual-task interference effects: performance on a task is usually worse when it is performed at the same time as another task compared to when that task is performed alone [12]. Such dual-task interference effects often stem from the limits on our basic cognitive and perceptual abilities: we often cannot actively engage in two tasks at the same time, but instead must interleave our efforts between tasks (e.g., [2,13–19])

For example, a driver who is writing a text message on a phone must take his or her eyes off the road to perform the text-typing task. However, this gives the driver a strategic choice. Should the driver write the entire text message at once and so take his or her eyes off the road for a long period of time? This might seem like a reckless decision. Alternatively, a few characters might be typed and attention returned to driving before a few more characters are typed. The choice of interleaving strategy has implications for how well each task is performed, giving a dual-task tradeoff (e.g., [14,20–25]). The person must decide which task is more important and so prioritize performance of one task over the other.

The focus of this paper is on understanding how people make dual-task interleaving tradeoffs. In doing so we seek to understand how good people are at multitasking. To address this question we report the results of an experiment in which participants had to perform two separate tasks at the same time but could only work on one task at a time. Participants therefore had to decide when to switch between tasks. Results show how this decision is systematically influenced by three factors: task characteristics, incentives, and individual differences in skill. Before describing the details of this study, and a computational model that was developed to understand the results, we first review work of each of the primary factors of interest.

### Task Characteristics

Previous work has extensively investigated how task difficulty affects multitasking performance (e.g., [21,26–28]). A theoretical interest has been in understanding the general characteristics that makes tasks hard to perform in multitasking settings. Two characteristics have been identified. First, task characteristics place limitations on performance, as the task in part dictates how fast a participant can complete its components (referred to as data-limitations in [27]). For example, a text message will be faster written on a phone that auto-completes words compared to a phone that does not auto-complete words, as in the first case less time is spent on typing each individual word. How difficult it is to combine tasks in a multitask setting also depends on the amount of overlap between the cognitive resources that are needed for the tasks [29–31]; the larger the overlap between resources that are needed for both tasks (e.g., vision, memory), the more difficult it is to perform tasks concurrently.

In our previous work investigating multitasking behavior we have used a tracking and typing task [18,32], which is inspired by the dialing-while-driving scenario described in the introduction. In our set-up, participants interleave between a typing task and a tracking task (described in more detail later) in a discretionary way (cf. e.g., [13,16,18,32]). That is, participants can only see and work on one task at a time and need to decide when to switch between tasks. The benefit of this discretionary set-up is that it gives a quick and easy way to directly infer the participant’s task interleaving strategy. However, a disadvantage of our discretionary set-up is that explicit switching between windows is relatively costly, requiring the participant to press a button on a joystick. There has been extensive discussion within the literature on how such 'information access-costs' can influence the emergence of interactive behavior (e.g., [33–39]). Eye-tracking has been successfully used in some previous studies to infer dual-task interleaving strategies, for instance see work by Hornof and colleagues [40,41].

In our analysis of optimality of the chosen strategy, we craft a model which also incurs these switch-costs and which is used to investigate the performance of various discrete interleaving strategies. This includes extreme strategies, ranging from a no-interleaving strategy, which does the typing task without checking on the tracking task even once, to a maximum interleaving strategy in which checks are made on the tracking task after entering each and every digit in the typing task. As such, our investigation covers the full range of possible task interleaving strategies and it is expected that performance will fall within these performance 'brackets' (cf. [42,43]).

We will now describe the two tasks, tracking and typing, in more detail. Variations in the characteristics of each task can influence how people choose to interleave attention when multitasking.

Tracking tasks have been used in various multitasking studies (e.g., [31,40–42,44–47]), and the difficulty of this task can be easily manipulated. In our tracking task, a moving cursor (10x10 pixels) needs to be kept inside a circular target area. We can manipulate two factors to control the difficulty of the tracking task: the radius of the target area and the function that controls the movement of the cursor. The target area has a radius of either 80 pixels (small radius) or 120 pixels (large radius). Keeping the cursor inside a small radius requires more frequent attention to the task than keeping the cursor inside the large radius. This is comparable with how it might be easier to keep a car inside a wide lane compared to a narrow lane. We also manipulate the speed with which the cursor moves around at times when it is not actively controlled by the participant. The position then updates following a random walk with mean of 0 pixels and a standard deviation of 3 (low noise) or 5 pixels per update (high noise). When the cursor movement has a higher standard deviation, it becomes less predictable and needs more attention. This is comparable with how driving a car at a high speed on a busy multilane highway is far more demanding and will require higher levels of vigilance than driving at a slow speed along a quiet back road.

Digit typing tasks have also been used in multitasking research concerned with driver distraction [14,24,25,48]. Previous research has shown that the way in which digits are typed is influenced by how they are represented. If numbers are displayed or memorized in a chunked manner, people tend to interleave at the boundaries between chunks [14,24,25,48]. In our study we control for these patterns by presenting the to-be-typed digits visually, thereby not relying solely on memory of structured representations.

In addition, motor actions can also provide cues for the interleaving of digits. Specifically, if a number contains both sequences of repeating digits and sequences of different digits that require a finger movement, then there is a benefit to interleave at points where the finger had to be moved [24]. In the current study we control for this by using a limited set of three digits (1, 2, and 3) and by encouraging participants to use dedicated fingers for typing each digit. We randomized the frequency and positioning of each digit, with the added constraints that each digit occurred at least six times and that each digit did not occur more than three times in sequence. How many digits are dialed in a sequence is also influenced by the priorities that people set [14,24,25]. In our study we manipulate priorities through the use of monetary incentives, which are discussed next.

### Incentives

Incentives can influence performance by placing relatively more value on one task compared to the other. In this study we use an explicit objective payoff function to incorporate incentives. The payoff function translates performance on each of the individual tasks into a single monetary value and combines these values into a single total score that is reported to the participant. The participant can then use this information to try and maximize their score.

Payoff functions have been used frequently in empirical studies, particularly as a way to motivate participants [49–51]. More recently, payoff functions have been advocated as being useful in combination with computational cognitive models [18,32,52–56]. In a multitasking setting, the use of a payoff function has three advantages. First, it provides the experimenter and the participant with an objective criterion to assess optimal performance: optimal performance is that which leads to the highest payoff score. Second, performance on individual tasks might be expressed in different units (e.g., speed, accuracy) and it might not be trivial to assess how a 'loss' on one task should be traded-off against a 'gain' on another task (but see [14,24] for one way of doing this). A pay-off function avoids this problem, by explicitly specifying how performance translates into a single unit across tasks. Third, people are known to have difficulties with maintaining internal scales of variance [57], for example to assess the exact brightness of a light. Such internal scales are not required when scores are made explicit by a payoff function. Instead, the objective monetary score can be used to assess whether performance on a current trial was better or worse compared to performance in other trials.

We use payoff functions to investigate how flexible performance is. We manipulate the payoff functions between participants, such that more or less value is placed on each of the two tasks, and investigate how well participants adjust their behavior to the payoff functions. This can be seen as a systematic way of changing participants' priorities [18]. If participants are solely driven by the task characteristics and not by incentives, then such changes should have little effect on performance. That is, participants might then be expected to use "default" ways of interleaving tasks [31]. However, we suspect that people are sensitive to incentives.

In preceding work, participants experienced one payoff function and we analyzed how well they performed against that payoff function [18]. However, we have not investigated how well participants perform in cases where the payoff function changes. Here, we empirically test whether participants use different strategies, and have different performance, when different payoff functions are being used. We then compare human performance to predictions by a computational cognitive model to see whether participants achieved the best performance that was possible given their characteristics and the payoff function that they experienced.

### Individual Differences

We also investigate how performance is affected by individual differences in task skill. There is a growing appreciation in the multitasking literature that task skill can influence multitasking performance (e.g., [10,11,41,52]). One idea is that the better an individual is at performing a task in isolation, the more able they are to perform that task in a dual-task setting (e.g., see Chapter 6 in [58]). Applying this to our tracking-while-typing task, we might expect individual differences in how quickly and accurately a person can type digits. As will be demonstrated in the empirical section, this individual difference in typing skill was found to influence choice of interleaving strategy and dual-task performance. We refer to typing as a "skill" in that typing ability is developed through years of practice (cf. e.g., [59,60]). We did not expect that there would be acquisition or strong improvement of this skill during our experiment.

The performance on individual tasks can influence performance in dual-task settings if there is time pressure. For example, in our experiment the cursor cannot be controlled while participants are working on the typing task. During this time the cursor will drift, and participants eventually need to check again whether they need to correct the trajectory of the cursor. Given the same time window, very skilled typists might be able to type more digits per visit to the typing task compared to less skilled typists. This is indeed what we find in our empirical results. The faster typists type more digits per visit to the typing window, however, the average time that is spent in the typing window is not affected by typing skill (see results). Such subtle difference in skill can then also further affect performance. For example, in our task participants need to type a finite string of digits, and faster typists might be faster at completing this task than slower typists—thereby achieving a better score.

Other individual differences might also have occurred during our experiment. In particular, there might have been differences in how well participants could control the joystick. However, the experiment did not contain an independent session that could be used to assess joystick control. Although participants practiced with the control of the joystick during the single-task tracking trials, these sessions were not systematic enough to assess participants' general tracking ability. We therefore did not include tracking as a skill factor in the statistical analyses and the model.

### Overview

In the remainder of this paper we first describe a dual-task experiment and demonstrate how participants’ performance of these tasks is influenced by task characteristics, incentives, and individual differences in skill. We then describe a computational cognitive model that is used to make performance predictions for the range of plausible dual-task interleaving strategies. The model is calibrated to the constraints of the task, the incentives (payoff function), and the observed (single-task) typing speed of individual participants. The model is used to identify the optimal task interleaving strategy (i.e., the strategy that maximizes reward through the payoff function in each condition for each participant). By taking this approach we were able to directly compare how participants performed in the experiment against the prediction of their optimal dual-task performance. This allows us to ask, in a very precise way, whether people are good multitaskers or not.

## Typing-while-tracking experiment

Building on previous dual-task experiments [18,32], participants were required to divide their efforts between two concurrent tasks. One task was to type a string of twenty digits using a keyboard. In the other task, a randomly moving cursor needs to be kept inside a circular target area using a joystick. Both tasks were presented on the same display, but participants could only see and control one task at a time and so needed to decide when to switch their attention between these tasks. For each task it is possible to define a performance metric (i.e., speed at which the typing task is completed and how well the cursor is kept inside the target area). It is then possible to combine these separate task performance metrics into a single payoff function, thereby allowing the relative value of each task to be varied between different experimental conditions. Specifically, in one between-subjects condition ('speed'), the payoff function puts relatively more value on fast completion of the typing task. Whereas, in another condition ('accuracy'), the payoff function puts relatively more value on keeping a randomly moving cursor inside a target area. As we shall describe in detail below, these payoffs were used as a monetary incentive scheme to reward participants in the experiment.

### Method

#### Participants

Twenty-four students (nine female) from the UCL psychology participant pool took part for monetary compensation. All participants were 18 years of age or older (*M* = 24.3, *SD* = 6.6, Max = 46 years). Payment was based on how well each task was performed (details in the Design section). Total payment ranged from £5.00 to £13.03 (*M* = £8.72). The UCLIC Ethics Committee (the University College London Interaction Centre's ethical committee) approved the study (approval number Staff/0910/005) and written consent was obtained from each participant.

#### Materials

**Fig 1:**
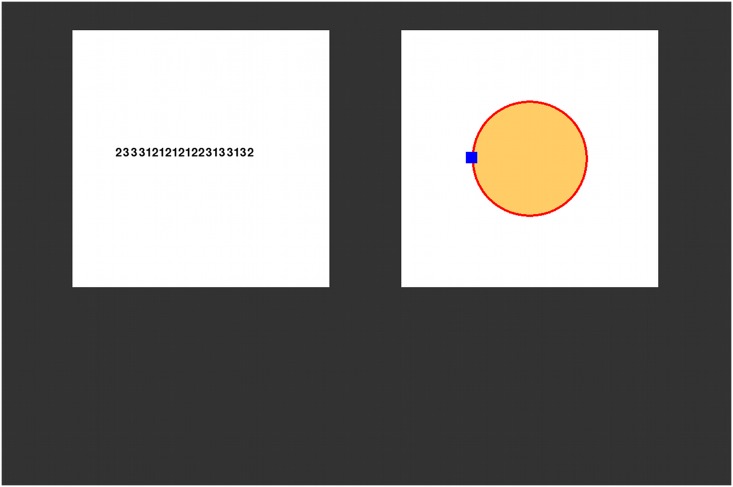
Layout of the tasks. Participants performed a typing task, which involved typing a string of 20 digits (left), and a tracking task (right), which involved keeping a blue cursor inside a circular target area (yellow). Participants could only see 1 task at a time and needed to determine when to switch their attention between tasks.

The dual-task setup was identical to that in [18] but differed in the payoff functions used. The experiment required participants to perform a continuous tracking task and a discrete typing task, presented on a 19-inch monitor with a resolution of 1280 x 1024 pixels (see Fig 1). The typing task was presented on the left side and the tracking task on the right. Each task was presented within a 450 x 450 pixels area, with a vertical separation of 127 pixels between the tasks.

The tracking task required participants to keep a moving square cursor (10 x 10 pixels) inside a target circle (see Fig 1). The target had a radius of either 80 (small radius) or 120 pixels (large radius). The movement of the cursor was defined by a random walk function. The rate of drift was varied between different experimental conditions. In a low noise condition, the random walk had a mean of zero and standard deviation of 3 pixels per update, while in a high noise condition the random walk had a mean of zero and standard deviation of 5 pixels per update. Updates occurred approximately once every 25 milliseconds. Participants used a Logitech Extreme 3D Pro joystick with their right hand to control the position of the cursor. The drift function of the cursor was suspended whenever the joystick angle was greater than +/- 0.08 (the maximum angle was +/- 1). The speed at which the cursor could be moved was scaled by the angle, with a maximum of 5 pixels per 25 milliseconds.

The typing task required participants to enter a string of twenty digits (chosen from digits 1 to 3) using a numeric keypad with their left-hand. Digits were presented in a randomized order with the constraint that no single digit was presented more than three times in a row in the sequence. A digit was removed from the string when it was entered correctly and all digits moved one position up. In this way, the left-most digit was always the next one to be entered. When an incorrect digit was typed, the string would not progress.

The study used a forced interleaving paradigm, in which only one of the two tasks was visible and could be worked on at any moment in time. By default the typing task was visible and the tracking task was covered by a gray square. Holding down the trigger of the joystick made the tracking task visible and covered the typing task. Releasing the trigger covered the tracking task and made the typing task visible once more. Participants could only control the task that was visible (e.g., the cursor would randomly drift and its position could not be corrected when it was not visible).

### Design

**Fig 2:**
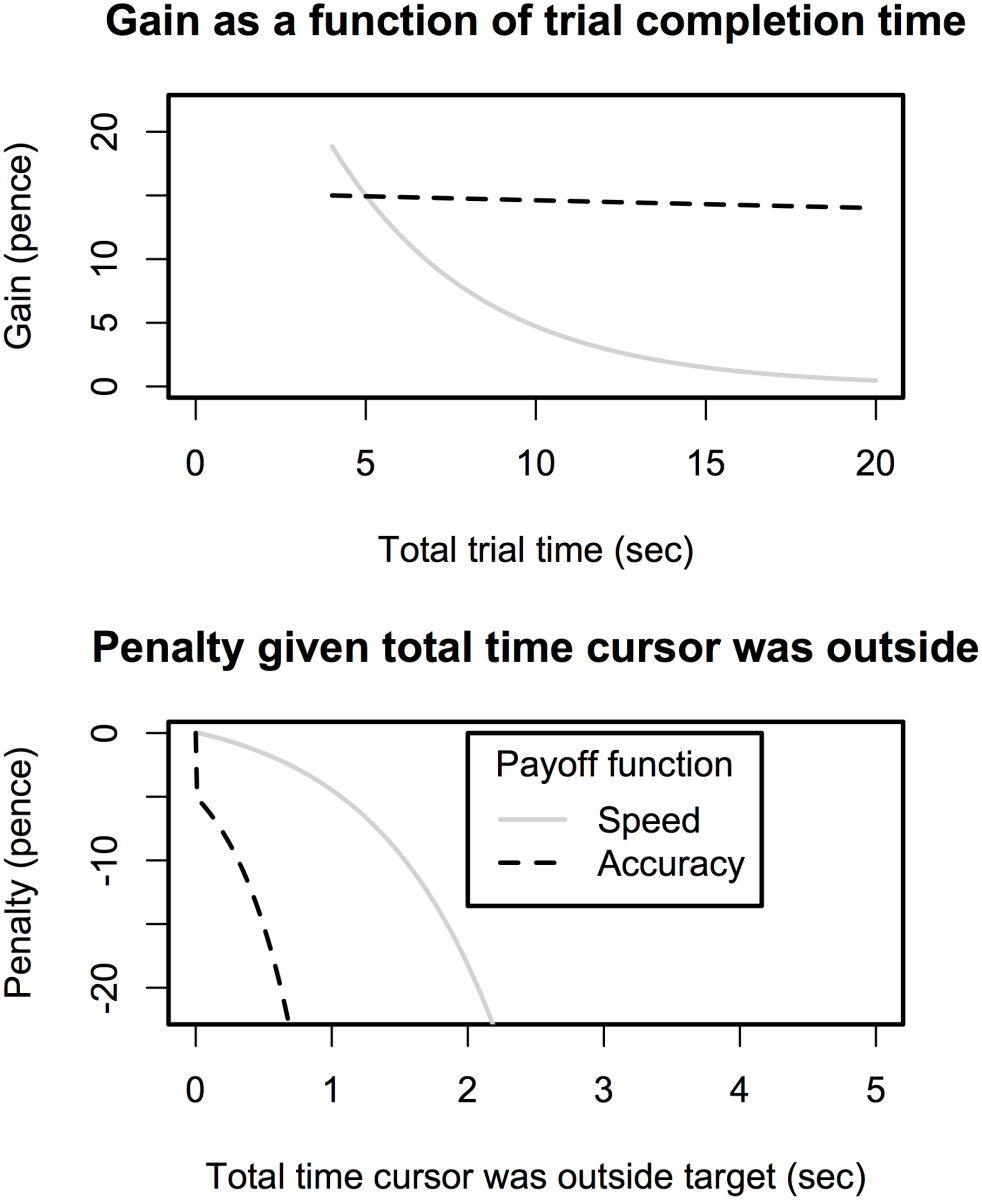
Illustration of how the two payoff functions affect score on each task. The top figure shows how score diminishes as total typing time increases. The bottom figure shows how score diminishes as the cursor spends more time outside of the target area. The Figure shows two lines, one for each payoff condition.

The experiment followed a 2 x 2 x 2 mixed factorial design. Within subjects, two factors of task characteristics were influenced: noise (high or low) and radius size (small or large). Between subjects, the payoff function was manipulated with 2 levels. Each payoff function adhered to the same basic structure (see below), but had different parameters so as to place different value on the typing and tracking task (see Table 1 for values and Fig 2 for an illustration). In both payoff conditions, both the speed of completing the typing task and the accuracy of performing the tracking task influenced the payoff score. However, between groups the relative weight of these two components differed. For ease of reference we therefore refer to the two groups as "speed" and "accuracy". In the 'speed' payoff condition, the parameters placed more weight on fast completion of the typing task, whereas in the 'accuracy' payoff condition more weight was placed on keeping the cursor inside the target area. Participants were randomly assigned to one or the other payoff condition in the experiment.

**Table 1:** Parameter values for the payoff function.

	Payoff function	
	Speed	Accuracy
**severityOfTrialTime**	-4.6209812	-0.0854888
**StartValue**_**gain**_	1.1552453	0.0170978
**compensation**	0.02294	0
**severityOfBeingOutside**	1.1090	2.2180
**startValue**_**tracking**_	1.5	0.6931

The payoff function had three components, as in Eq 1:

Payoff = Gain + Tracking Penalty + Digit Penalty (1)

Participants could *gain* points on the typing task, where faster trial times lead exponentially to higher scores, as in Eq 2:

$$\text{Gain }=\;0.15\;\times {\text{e}}^{{{}^{\text{severityOfTrialTime x }\left(\text{TotalTrialTimeInSeconds }/\;20\right)\;+\text{ startValue}}}_{{}_{\text{gain}}}}$$ (2)

That is, gain had an exponential relationship with the total time that was needed to complete the typing task (variable "TotalTrialTimeInSeconds"). Longer trial times lead to lower gain scores. To offset the impact, this score was multiplied with a parameter that could reduce the severity of longer trial times ("severityOfTrialTime") and the gain value was given a start value (startValue_gain_). Having a higher start value and a smaller value for the severity of trial time lead to higher gain scores. Table 1 provides the parameter values that were used in the two payoff conditions. The top figure in Fig 2 illustrates how the "gain" component of the score changed as a function of the total trial time. It can be seen that in the "speed" condition the decline in gain as a function of trial time is steeper.

A *digit penalty* of—£0.01 was applied for every digit that was typed incorrectly.

An exponential *tracking penalty* was applied when the cursor moved outside of the target area, as in Eq 3:

$$\text{Tracking Penalty}=\text{ compensation - }0.10\;\times \;{\text{e}}^{{}^{{}^{\text{SecOutside x severityOfBeingOutside }–\text{ startValu}{\text{e}}_{{}_{\text{tracking}}}}}}$$ (3)

The tracking penalty function has an exponential relationship with the total time that the cursor spent outside of the target area (parameter *SecOutside*). Longer times outside of the target area lead to stronger penalties. Again, this function was offset by a startvalue (*startValue*_*tracking*_) and multiplied with a parameter to reduce the impact of time outside (parameter *severityOfBeingOutside*). To avoid participants from losing all their money on a given trial, the payoff function had a minimum score of– £0.20. Table 1 provides the parameter values that were used in the two payoff conditions. Fig 2 illustrates how the tracking penalty accumulated as a function of time that the cursor was outside of the target area. In the "accuracy" payoff condition, the penalty increases more rapidly compared to the "speed" payoff condition.

### Procedure

Participants were informed that they would be required to perform a series of dual-task trials and that they would be paid based on their performance. A participant’s payment was based on the cumulative payoff over the course of the study, in addition to a base payment of £5 (participants in 'speed' payoff condition) or £3 (participants in 'accuracy' payoff condition). Different base payments were chosen, as the average gain per trial differed between conditions. By choosing a different base-rate, each participant had a guaranteed minimum payment of £5 (the institute's default payment rate per hour).

After an explanation of the task, participants performed two single-task training trials for each task and two dual-task practice trials. Participants were instructed that in dual-tasks they could only see and control one task at a time and had to actively switch between tasks by pressing the trigger button on the joystick.

Participants then completed four blocks of experimental trials (one for each experimental condition). In the first two blocks, participants experienced a single noise level, either low or high noise. The noise level was randomly assigned and balanced across participants. On the first block a radius size (small or large) was also randomly assigned, on the second block the other radius level was assigned. For the third and fourth block this order of radius conditions was repeated, but with another level for noise. For each block, participants completed five single-task tracking trials, five single-task typing trials, and twenty dual-task trials. The dual-task trials were further grouped into sets of five trials, with a short pause between each set. The total procedure took about one hour to complete.

Participants were aware that the payoff that they received was influenced by their performance on the typing task and by their performance on the tracking task. Specifically, in all conditions, participants were told that they could gain points by completing the typing task as quickly as they could and that faster trial completion times would lead to higher scores. All participants were also instructed that they lost points when the cursor went outside of the target area. They were also informed that they lost points when they made typing errors. Or to state differently: all participants were informed that both speed (on the typing task) and accuracy (on the tracking task) mattered. However, they were not informed of the exact equations that underlie their payoff, nor of the relative weight of each component (i.e., whether fast completion or tracking accuracy were more valuable). This allowed us to investigate how well participants adapted their performance to the feedback they received on their performance at the end of each trial. Do people behave differently based on the payoff function, or do they apply "default" interleaving strategies that are independent of the payoff function? For ease of reference, we refer to our two groups of participants as "speed" and "accuracy" to emphasize what task had a relatively stronger weight in the payoff function. However, both aspects mattered in both payoff conditions.

### Measures

In our main analysis we report results only for the last 5 trials of each block. The motivation for this is that we are interested in participants’ behavior after they had time to become accustomed to the payoff function and have received feedback on their performance. For each metric we calculate a score (e.g., total trial time) per trial and report the average score across the 5 trials. This average score is also used in statistical analyses.

Performance is expressed in three metrics: total trial time, maximum deviation of the cursor from the center of the target area, and total time the cursor spent outside of the target area. Total trial time is defined as the time between the start of the trial and the time at which the last digit of the string of digits was pressed.

For maximum deviation of the cursor we calculated per trial what the furthest deviation of the cursor from the center of the target was. For each participant we then calculated the average value across trials. This measure is of interest given its similarity to a metric of driver distraction research: how far does a car (here: cursor) drift outside of the lane boundary (here: target area) due to inattention?

The third measure is the average total time that the cursor spent outside of the target area. The metric is again related to measures of driver distraction: how long was a car (here: cursor) outside of the lane boundary (here: target area) due to inattention?

We also analyzed four related metrics that reflect participants' strategy for interleaving between tasks. The maximum number of digits typed per visit to the typing window reflects how long participants were willing to stay in the typing window while the cursor drifted out of sight. Only correctly typed digits were considered. The second metric is the average time that was spent per visit to the typing window. The third metric is the average number of visits to the tracking window. The fourth metric is the average time that is spent in the tracking window per visit. Taken together, these four metrics describe how frequently participants visit each task and how long they spend on each task before moving on to the next task. This again relates to measures of driver distraction that investigate how frequently and how long participants glance at the road (here: number of visits to tracking task and duration of that visit) and how much time they spend on a distracting task (here expressed as maximum number of steps completed and as average visit time).

In our analysis we found that participants differed in their typing speed and that this affected performance and strategy. To incorporate this into our statistical analysis, we split the participants of each payoff condition into two groups using a split mean procedure on the average interkeypress interval times (IKI). This resulted in four equal groups: fast typers in the speed payoff condition (IKIs of 184, 184, 198, 264, 286, and 309 msec), slow typers in the speed payoff condition (IKIs of 317, 382, 384, 394, 394, and 470 msec), fast typers in the accuracy payoff condition (IKIs of 211, 224, 226, 255, 259, and 276 msec), and slow typers in the accuracy payoff condition (IKIs of 290, 388, 403, 405, 443, and 451 msec).

For statistical analysis we used a 2 (payoff function: speed/accuracy) x 2 (cursor noise: low/high) x 2 (target size: small, large) x 2 (typing speed: relatively slow/fast) ANOVA. We only considered main effects and two-way interactions. A significance level of .05 was applied throughout. Table 2 gives an overview of the statistical effects found. These are discussed in more detail in the text.

**Table 2:** Summary of statistical effects in the experiment.

	Dependent variable						
	Total trial time	Maximum cursor deviation	Total time cursor outside target	Maximum nr of digits per visit	Mean visit time, typing window	Nr visits to tracking task	Mean visit time, tracking window
**Payoff function (P)**		**	.	***	***	**	
**Noise (N)**	***	***	***	***	***	***	***
**Radius (R)**	***	***	***	***	***	***	***
**IKI group (I)**	***	***	**	***		**	*
**P x N**		**	*	.	*		
**P x R**						*	
**N x R**	*	*	**				
**P x I**				*			
**N x I**	*		.				
**R x I**	*		**			.	

.: .05 < p < = .10;

*: .01 < p < .05;

**: .001 < p < .01;

***: p < = .001

## Results and Discussion

### Overall performance

**Fig 3:**
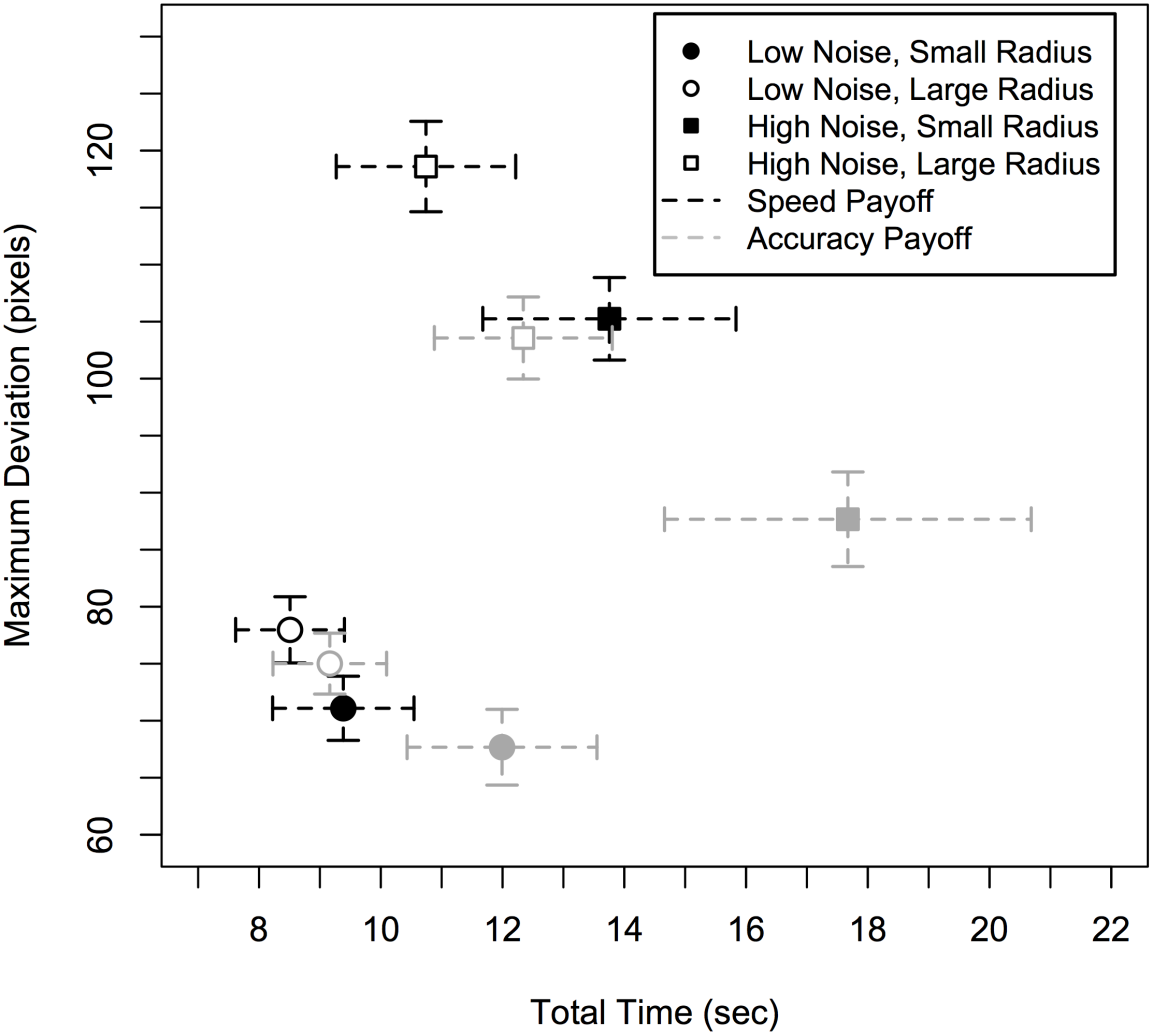
Plot of the performance trade-off space for all eight conditions. Data shows total time against maximum deviation of the cursor. Error bars show standard errors.

Fig 3 plots the performance space of total trial time versus the maximum distance that the cursor moved away from the center of the target in one plot for all eight conditions. The majority of the eight conditions roughly take up a unique point in this performance space, suggesting that performance was different in each condition.

In general, the cursor deviated more for the 'speed' (of typing) payoff condition (Fig 3: black points) than for the 'accuracy' (of tracking) condition (grey points). The cursor also deviated more when the noise was high (squares) compared to low (circles), and when the radius was large (open points) compared to small (closed points). For trial time, performance was mostly affected by task difficultly, as trial times were shorter when noise was low (circles), or when the radius was large (open points). Statistical analysis confirmed these findings. The effects are summarized in Table 2, and discussed in more detail below. The raw data is included together with an R analysis script in S1 file.

Trial time was affected by the characteristics of the task. Specifically, the trial time was longer when there was high noise (*M* = 13.62 sec, *SD* = 6.71 sec) compared to low noise (*M* = 9.76 sec, *SD* = 3.89 sec), *F*(1, 21) = 26.38, *p* < .001, η_p_^2^ = 0.557. Similarly, trial time was longer when the radius of the target was small (*M* = 13.20 sec, *SD* = 6.54 sec) compared to large (*M* = 10.19 sec, *SD* = 3.99 sec), *F*(1, 21) = 21.93, *p* < .001, η_p_^2^ = 0.511. That is, people were slower when the task conditions were more difficult. Total trial time was also affected by typing speed, *F*(1, 20) = 31.68, *p* < .001, η_p_^2^ = 0.613. Perhaps not surprisingly, participants that were faster at typing had a shorter trial time (*M* = 7.87 sec, *SD* = 1.74 sec) compared to those that were slower at typing (*M* = 15.52 sec, *SD* = 4.47 sec). That is, trial time was almost twice as short for those that were faster at typing compared to those that were slower. Surprisingly, there was no main effect of payoff function. There were also significant two-way interaction effects. The level of noise on the tracking task interacted with interkeypress interval group, *F*(1, 21) = 4.66, *p* = .043, η_p_^2^ = .181. Target radius also interacted with interkeypress interval group, *F*(1, 21) = 5.03, *p* = .036, η_p_^2^ = .193. Finally, there was an interaction between cursor noise and target radius, *F*(1, 23) = 4.893, *p* = .037, η_p_^2^ = .175. There were no other significant effects.

For maximum deviation, the cursor deviated more in the speed payoff condition (*M* = 93.23 pixels, *SD* = 7.84 pixels) compared to the accuracy payoff condition (*M* = 83.48 pixels, *SD* = 8.77), *F*(1, 20) = 14.55, *p* = .001, η_p_^2^ = .421 That is, when the penalty for being outside of the target area was harsher (accuracy condition), participants kept the cursor closer to center. Not surprisingly, the cursor also deviated more in the high noise condition (*M* = 103.77 pixels, *SD* = 13.76 pixels) compared to the low noise condition (*M* = 72.93 pixels, *SD* = 8.21 pixels), *F*(1, 21) = 200.334, *p* < .001, η_p_^2^ = .905. The cursor also deviated more in the large radius condition (*M* = 93.78 pixels, *SD* = 10.53 pixels) compared to the small radius condition (*M* = 82.92 pixels, *SD* = 11.00 pixels), *F*(1, 21) = 29.50, *p* < .001, η_p_^2^ = .584. The cursor also deviated more for slow typers (*M* = 93.85 pixels, *SD* = 8.74 pixels) than for fast typers (*M* = 82.85 pixels, *SD* = 6.92 pixels), *F*(1, 20) = 18.55, *p* < .001, η_p_^2^ = .481. There were two significant interaction effects. First, payoff function interacted with noise level, *F*(1, 21) = 9.07, *p* = .007, η_p_^2^ = .302. Second, noise and radius interacted, *F*(1, 23) = 5.00, *p* = .035, η_p_^2^ = .179. There were no other significant effects.

For total time that the cursor spent outside of the target area, there was a marginal effect of payoff function, *F*(1, 20) = 4.12, *p* = .056, η_p_^2^ = .171, such that mean time that the cursor was outside of the target area was longer in the speed payoff condition (*M* = 0.57 sec, *SD* = 0.34 sec), compared to the accuracy payoff condition (*M* = 0.33 sec, *SD* = 0.32 sec). The total time outside was also affected by the task difficulty. The cursor was longer outside of the target area in the high noise condition (*M* = 0.74 sec, *SD* = 0.63 sec) compared to the low noise condition (*M* = 0.16 sec, *SD* = 0.16), *F*(1, 21) = 26.616, *p* < .001, η_p_^2^ = .559. The cursor was also longer outside of the target area when the radius was small (*M* = 0.71 sec, *SD* = 0.58 sec) compared to when it was large (*M* = 0.19 sec, *SD* = 0.19 sec), *F*(1, 21) = 34.41, *p* < .001, η_p_^2^ = .621. Finally, the time outside was also affected by typing speed, *F*(1, 20) = 10.08, *p* = .005, η_p_^2^ = .335. The time outside of the target area was almost twice as long for slow typers (*M* = 0.63 sec, *SD* = 0.37 sec) compared to fast typers (*M* = 0.27 sec, *SD* = 0.20 sec). These patterns were affected by three interaction effects, namely between payoff function and noise (*F*(1, 21) = 4.64, *p* = .043, η_p_^2^ = .181), between noise level and radius (*F*(1, 23) = 8.86, *p* = .007, η_p_^2^ = .278), and between radius and typing speed (*F*(1, 21) = 10.62, *p* = .004, η_p_^2^ = .336). Finally, there was a marginal significant interaction effect between noise level and interkeypress interval group, *F*(1, 21) = 3.211, *p* = .088, η_p_^2^ = .133. There were no other significant effects.

Taken together, the analysis shows that the difficulty of the tracking task (i.e., noise and radius) consistently affected performance on each task. Similarly, individual difference in participants’ typing speed affected performance on each task. Manipulation of the dual-task payoff function had an effect on how participants performed on the tracking task (i.e., maximum cursor deviation and total time that the cursor was left outside of the target). More specifically, participants tended to allow the cursor to drift further, and let it remain outside of the target area for longer, when the payoff function rewarded faster completion of the typing task compared to accurate tracking performance. However, there was no effect of payoff manipulation on total trial time. To better understand these results, we next consider metrics related to how participants choose to interleave tasks.

### Dual-Task Interleaving Strategies

**Fig 4:**
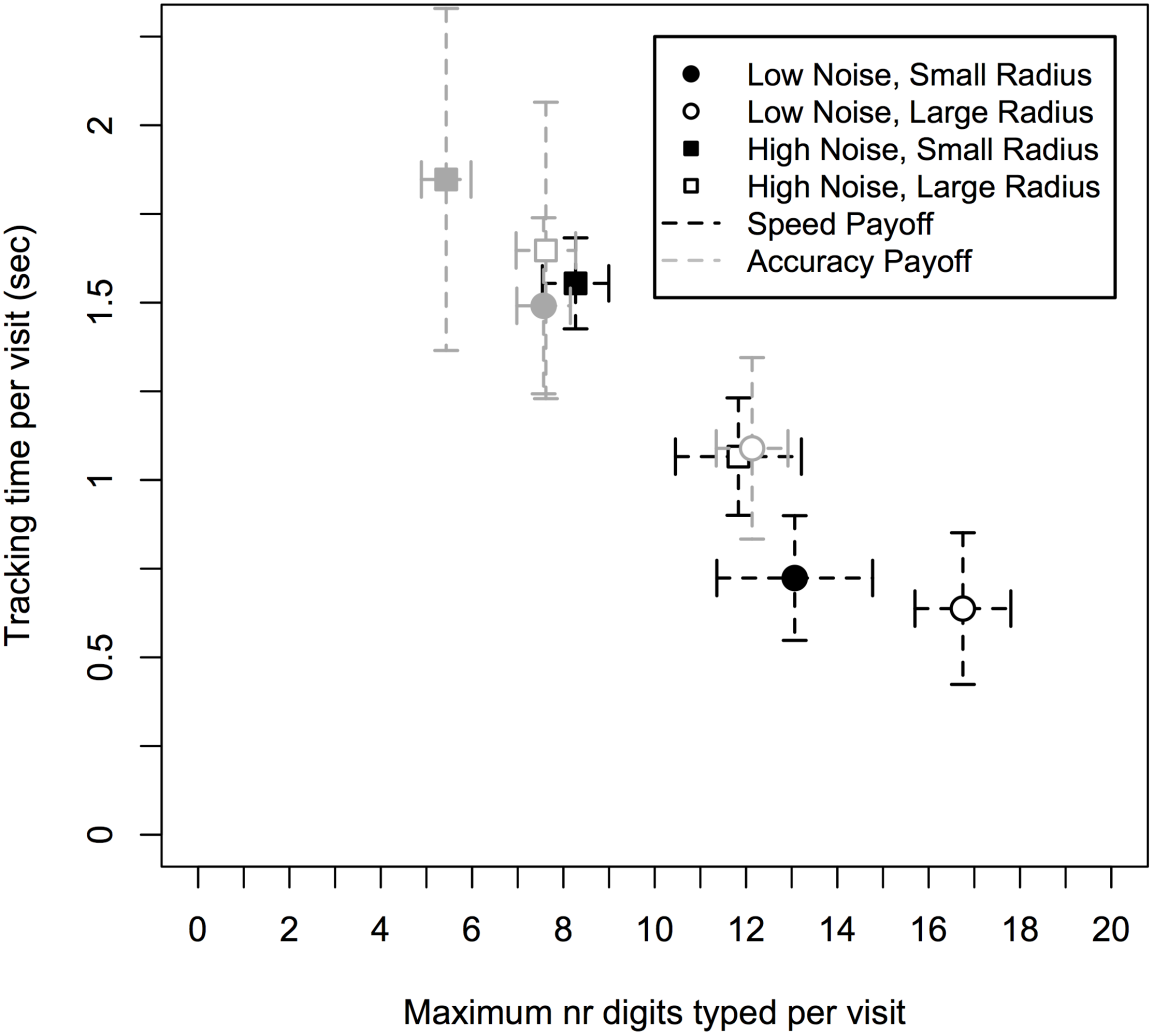
Plot of the strategy trade-off space. Data shows maximum number of digits typed and time spent tracking per visit. Error bars show standard errors.

Fig 4 plots two measures of dual-task interleaving strategy: the maximum number of digits that participants’ choose to type during a visit to the typing window, and the duration of time that was spent in the tracking window per visit to this window. Again, each experimental condition has a relatively unique point in this strategy space, especially when comparing the two payoff conditions (i.e., compare the black with the grey points in Fig 4). A summary of statistical effects is given in Table 2, and discussed in more detail below.

For the maximum number of digits typed per visit to the typing window, more digits were typed in the speed payoff condition (*M* = 12.48 digits, *SD* = 3.67 digits) compared to the accuracy payoff condition (*M* = 8.19 digits, *SD* = 1.33 digits), *F*(1, 20) = 29.56, *p* < .001, η_p_^2^ = 0.596. That is, more digits were typed when the payoff condition encouraged fast completion of the typing task (speed payoff condition). The maximum number of digits was also affected by task characteristics, such that more digits were typed per visit to the typing window when the task environment conditions were easier (i.e., low noise, large radius). Specifically, more digits were typed when noise was low (*M* = 12.38 digits, *SD* = 4.03 digits), compared to when noise was high (*M* = 8.29 digits, *SD* = 3.25 digits), *F*(1, 21) = 85.26, *p* < .001, η_p_^2^ = 0.802. More digits were also typed when the radius was large (*M* = 12.08 digits, *SD* = 3.53 digits) compared to when the radius was small (*M* = 8.58 digits, *SD* = 3.69 digits), *F*(1, 21) = 73.11, *p* < .001, η_p_^2^ = 0.777. The number of digits was also affected by typing speed, *F*(1, 20) = 17.39, *p* < .001, η_p_^2^ = 0.465. Fast typers typed more digits per visit to the typing task (*M* = 11.98 digits, *SD* = 4.07 digits) than the slow typers (*M* = 8.69 digits, *SD* = 1.67 digits). Two interaction effects further influenced these results. There was a significant interaction between payoff function and interkeypress interval group, *F*(1, 20) = 7.53, *p* = .012, η_p_^2^ = .273, and there was a marginal significant interaction effect between noise level and interkeypress group, *F*(1, 21) = 2.993, *p* = .098, η_p_^2^ = .125. There were no other significant effects.

That participants chose different strategies for the maximum number of digits is also reflected in the average time that they spent in the typing window per visit. More time per visit was spent in the speed payoff condition (*M* = 3.31 sec, *SD* = 0.80 sec) compared to the accuracy payoff condition (*M* = 2.03 sec, *SD* = 0.53 sec), *F*(1, 20) = 20.86, *p* < .001, η_p_^2^ = .511. That is, more time per visit was spent on the typing task when the payoff function weighed fast completion of the task more strongly. The time was also affected by the task difficulty, such that shorter visits were made when the tracking task was harder (e.g., due to a small radius or high noise). Visit times to the typing window were shorter for the high noise condition (*M* = 1.95 sec, *SD* = 0.68 sec) compared to the low noise condition (*M* = 3.39 sec, *SD* = 1.38 sec), *F*(1, 21) = 44.30, *p* < .001, η_p_^2^ = .678. Visit times to the typing window were also shorter for the small radius condition (*M* = 2.19 sec, *SD* = 0.98 sec) compared to the large radius condition (*M* = 3.14 sec, *SD* = 1.19 sec), *F*(1, 21) = 17.62, *p* < .001, η_p_^2^ = .456. Finally, there was an interaction effect between payoff function and noise level, *F*(1, 21) = 5.117, *p* = .034, η_p_^2^ = .196. There were no other significant effects. Specifically, there was no significant effect of typing speed. When comparing these results with the analysis of maximum number of digits typed per visit, the lack of a significant effect of typing speed on mean visit time to the typing window suggests that participants had set an objective criterion for how long they could spend in the typing task and that this criterion depended on the payoff condition and the task difficulty. Given this criterion, a participant can type more or less digits depending on their typing skill—but still spends roughly the same time per visit independent of typing skill.

These differences in the length of each visit to the typing task and in the maximum number of digits typed per visit to the typing task also affected how often participants visited the tracking task. Participants made more visits to the tracking window when the payoff function promoted accuracy (*M* = 3.59 visits, *SD* = 1.70 visits) compared to when it promoted speed (*M* = 2.01 visits, *SD* = 1.38 visits), *F*(1, 20) = 9.05, *p* = .007, η_p_^2^ = .311. The number of visits was also affected by task difficulty. More visits were made when noise was high (*M* = 3.79 visits, *SD* = 2.52 visits) compared to when noise was low (*M* = 1.81 visits, *SD* = 1.21 visits), *F*(1, 21) = 25.32, *p* < .001, η_p_^2^ = .547. More visits were also made when the radius of the target area was small (*M* = 3.63 visits, *SD* = 2.40 visits) compared to when it was large (*M* = 1.98 visits, *SD* = 1.14 visits), *F*(1, 21) = 35.92, *p* < .001, η_p_^2^ = .631. Finally, more visits were made by slow typists (*M* = 3.71 visits, *SD* = 1.65 visits) compared to fast typists (*M* = 1.89 visits, *SD* = 1.27 visits), *F*(1, 20) = 12.15, *p* = .002, η_p_^2^ = .378. These results were further influenced by two interaction effects: a significant interaction between payoff function and radius (*F*(1, 21) = 5.15, *p* = .034, η_p_^2^ = .197) and a marginally significant interaction between radius and typing speed (*F*(1, 21) = 3.41, *p* = .079, η_p_^2^ = .140.

Finally, we also analyzed the average time spent in the tracking window per visit to this window. This time was affected by task difficulty, such that more time was spent in difficult situations (e.g., small radius, high noise). More time was also spent in the tracking window when noise was high (*M* = 1.53 sec, *SD* = 1.14 sec) compared to when noise was low (*M* = 0.99 sec, *SD* = 0.80 sec), *F*(1, 21) = 16.13, *p* < .001, η_p_^2^ = .434. More time was also spent in the tracking task per visit when the radius was small (*M* = 1.40 sec, *SD* = 0.96 sec) compared to when the radius was large (*M* = 1.11 sec, *SD* = 0.92 sec), *F*(1, 21) = 19.82, *p* < .001, η_p_^2^ = .486 Surprisingly, the time spent in the tracking window per visit also changed with typing speed, *F*(1, 20) = 5.36, *p* = .031, η_p_^2^ = .211. Slow typers spent more time in the tracking window (*M* = 1.66 sec, *SD* = 1.14 sec) compared to fast typers (*M* = 0.86 sec, *SD* = 0.40 sec). This result might be due to a floor effect: some fast typers could complete the typing task without ever visiting the tracking window in some of the conditions (e.g., large radius with low noise). In contrast, the slow typers always had to visit the tracking task. This might have made their average time on the tracking task (i.e., the main effect of typing group) slightly higher.

Taken together, the above analyses show that participants’ dual-task interleaving strategy was affected by the three factors of interest: changes to the payoff function, changes to the difficulty of the tracking task (noise and radius), and individual differences in participants’ typing speed. For example, participants dedicated more of their time to the typing task and paid fewer visits to the tracking task when the payoff function rewarded fast completion of the typing task more strongly. Similarly, when the tracking task was easier (i.e., when the cursor moved slower and the target was larger), visit times to the typing tasks were longer and fewer visits were made to the tracking task. Typing speed only affected some metrics. For example, it did not influence how long each visit to the typing task was, but it did influence how productive each visit was: fast typers completed more of the letter string than slow typers in the same time window.

## Discussion of results

The results of this experiment show what performance metrics and dual-task interleaving strategy were affected by our three factors of interest: task characteristics (noise, radius), individual differences in skill (typing speed), and incentives (payoff function). What these data do not reveal is whether participants adopted strategies that would result in the highest possible monetary reward over the trial—given the constraints that these factors place on performance. To better understand this aspect of the data we developed a computational cognitive model of task performance. The model is used to explore the performance of various dual-task interleaving strategies so as to identify the range of strategies that would yield the highest possible reward, given the constraints imposed on performance by the task (e.g., cursor noise, radius size, payoff function) and the individual (e.g., typing speed).

## Model

### Model development

Our model of dual-task performance is a modification of the model of average performance in [18]. The refinements are that the current model can capture individual differences in typing speed and can account for typing errors. A detailed description of model development and parameter choices is given in [54]. The model is used to predict performance for various strategies for interleaving attention between tasks.

The model captures each task (typing, tracking) as a series of discrete steps. This is similar to other procedural models of dual-task performance (e.g., [31,41]). However, compared to the preceding models, we model actions at the keystroke level (cf., [43,61]) and don't make strong assumptions about actions at the millisecond level. This level of abstraction has been valuable in other dual-task models [14,18,24,32].

We refer to our model as a 'computational cognitive model'. The term "cognitive" is used in reference to Newell's definition of the "cognitive band" of cognition ([62], see also [63]). Newell describes different types of human behavior that take place over different time scales (i.e., ranging from microseconds to months or years). Within this framework the 'cognitive band' takes place between a few hundred milliseconds to several seconds. Similarly, our model captures behavior that takes place at this timescale by specifying actions that take several hundreds of milliseconds (the keystroke level, cf. [43,61]). We call our model a computational model, as it is implemented as executable code; which is distinct from Marr's notion of computational explanation [64]. We will now describe the structure of the model in more detail.

### Typing model

The typing model types in digits according to a pre-specified strategy that is set by the modeler (see section on Strategy space below). The typing speed is calibrated to each individual participant’s average interkeypress interval as measured in single-task trials.

The model also makes typing errors, at the same rate as individual participants in single-task typing trials. Errors are inserted at random positions in the string of digits on each model run. It was assumed that typing an erroneous digit required the same time as a correct digit. In addition, it was assumed that a post-error slowing cost [65] slowed down typing speed on the immediately following correct digit. The mean post-error slowing time was estimated by subtracting the normal interkeypress interval time from the average time observed in the interval for the first correct digit after an erroneous digit. This model captures the core features of interest and is sufficient for making detailed predictions of typing time across a range of different dual-task interleaving strategies.

### Tracking model

The tracking model focuses on two core aspects of the experimental task: (1) that the cursor can only be controlled when the tracking window is open, and (2) whenever the cursor is not controlled it drifts according to the drift function of the experiment (see Methods section). At times when the model controlled the cursor movement, this was done as follows. Every 250 msec the position of the cursor relative to the center of the target area was determined. A linear function was then used to determine the angle of the joystick to move the cursor towards the center (this function was determined in [18]):

 Angle = -0.01 * current distance from target center          -1 <= angle <= 1

Based on the angle, the position of the cursor was updated every 25 msec by multiplying the angle value with 5 pixels. Both the frequency of the update and the angle multiplication were identical to how this was implemented in the experiment.

### Dual-task model

On each trial, the dual-task model typed a series of digits using the typing model before switching to the tracking task. The number of to-be-typed digits was specified as an explicit strategy choice. When the model switches between typing and tracking, a switch cost was incurred (250 msec, taken from [18]). The model then pursued active tracking of the cursor, based on the tracking model for a pre-determined fixed period of time. After this time had passed, another switch cost was incurred (180 msec, taken from [18]). The higher switch cost to switch from typing to tracking intuitively reflects the need to first locate the cursor on the screen—the digits are always in the same position and therefore require less time to locate.

Once the model switched back from tracking to typing, it would continue typing until it was time to switch again. It would continue this pattern until all 20 digits were typed in correctly.

### Strategy Space

We explored how different explicit strategies for interleaving tasks affected performance. A strategy was determined by two variables (1) a basic strategy determined how many digits were typed per visit to the typing window before switching to the tracking task, and (2) a strategy alternative determined how much time was spent in the tracking window on each visit before switching back to the typing task.

For the basic strategies (number of digits typed per visit), we explored performance for a relatively simple set of twenty strategies in which a consistent number of digits was typed per visit to the typing window. For example, a strategy to always type 1 digit per visit would make twenty visits; a strategy to always type 2 digits per visit would make ten visits; a strategy to always type 8 digits per visit would make two visits in which 8 digits were typed and one in which the remaining 4 digits were typed.

For each of these twenty basic strategies, we explored the performance of various strategy *alternatives*. Strategy alternatives varied in how much time was spent in the tracking window per visit to this window. We explored this for 12 alternatives, between 250 and 3,000 msec, in steps of 250 msec. Within a single simulation we kept the time spent in the tracking window per visit constant (i.e., if the model spent 250 msec during the first visit in the tracking window, a similar time was used the second visit).

For each of these distinct strategy variants the model was run multiple times and performance predictions were made. In total this lead to the use of 229 strategy alternatives. For 19 strategies (typing 1 to 19 digits per visit), we explored the effect of 12 alternatives for time on the tracking task (giving 12 x 19 = 228 strategy alternatives). There was one strategy without interleaving (typing all 20 digits in one visit). We ran 50 simulations (i.e., 50 simulated trials) for each individual, each experimental condition (noise, radius, payoff), and each strategy alternative. This gave a total of 12 (participants per payoff function) x 2 (payoff functions) x 2 (noise) x 2 (radius) x 229 (strategy alternative) x 50 (simulations) = 1,099,200 simulations. For each model simulation we were able to derive performance measures equivalent to those gathered for human participants (i.e., total trial time, maximum deviation of the cursor, total time that the cursor spent outside of the target area). Given these performance measures it was possible to calculate the payoff achieved by the model on each simulated trial using the same objective function for rating human performance in the experiment (see Eqs 1–3).

## Model Results and Discussion

### Comparison of human performance with predicted optimal performance

The empirical results demonstrated that participants adapted their strategies to the payoff function, the task characteristics, and their individual typing skill. With the model, we now want to ask a different question, namely: were participants good multitaskers?

To address this question, we selected for each individual participant, in each experimental condition, the strategy alternative that, on average, was predicted to achieve the highest payoff. We compared performance of this strategy on various metrics with human performance (as reported above). For some individuals, in some conditions, the model predicted that multiple strategies could achieve the highest score (i.e., no one strategy alternative was better than *all* other strategy alternatives). In these cases, performance for all measures of interest (e.g., trial time, maximum deviation of the cursor, number of digits typed per visit) was averaged across the set optimal strategies. This method allowed for a comparison between model and data without additional assumptions about how participants might choose between strategies that are otherwise equivalent in terms of their expected payoff. For example, alternative selection methods might be to 'bracket' the range of good performance [42,43] or to select the strategy that achieved the best mean value on some other measure of performance (e.g., trial time, or maximum deviation of the cursor). This would require additional assumptions about what the most representative/best metric is. Our approach does not require such additional assumptions.

**Fig 5:**
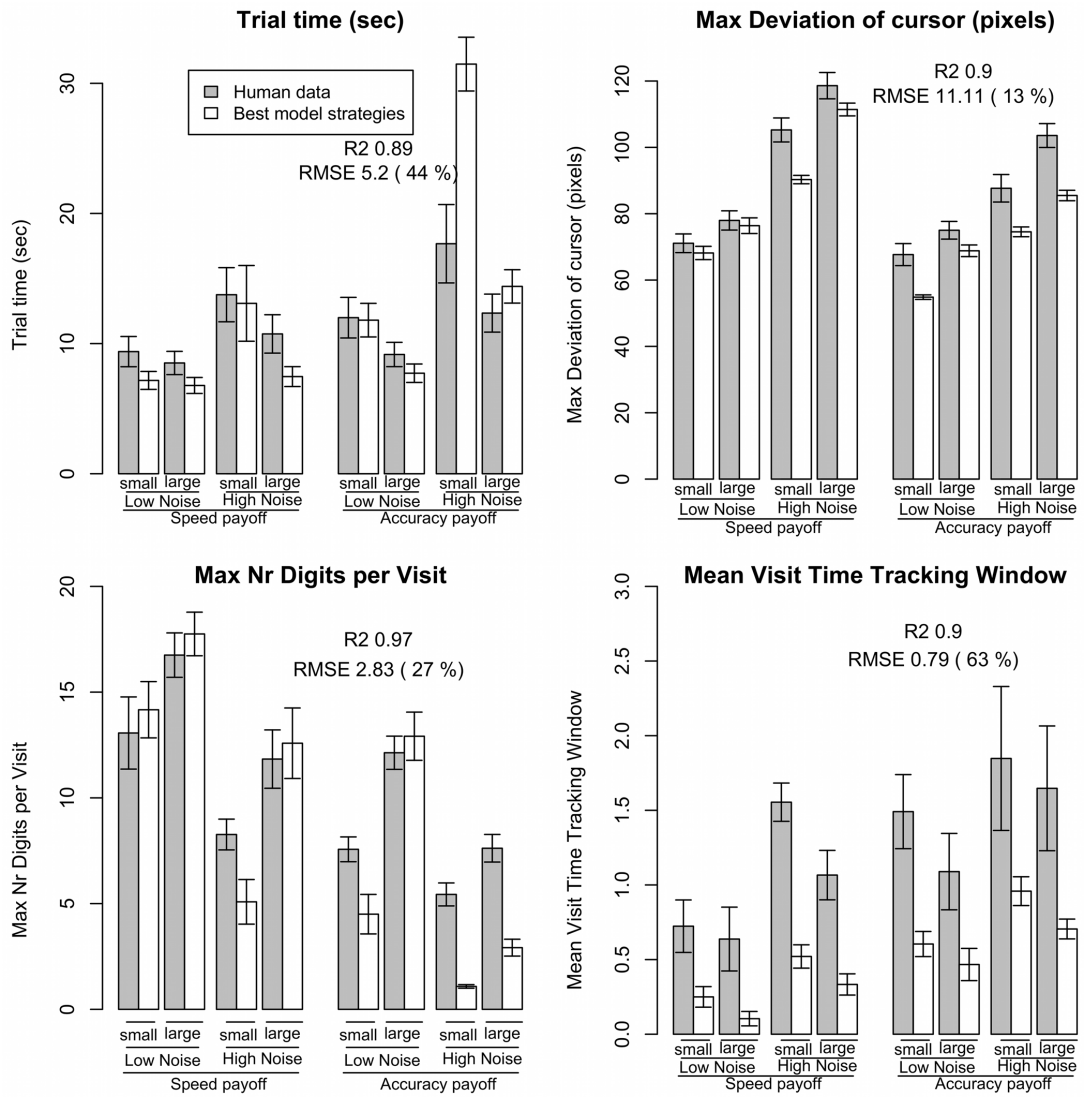
Correspondence between human mean performance and model predictions of the optimal strategies. Data shown for (top-left) total trial time, (top-right) maximum deviation of the cursor, (bottom-left) maximum number of digits typed per visit to the typing window, and (bottom-right) average time spent in the tracking window per visit. Error bars show standard errors.

Fig 5 shows the performance for the model (white bars) and human data (grey bars) side by side for four measures: total trial time (top-left), maximum deviation of the cursor (top-right), average maximum number of digits typed per visit (bottom-left), and mean time spent in the tracking window per visit (bottom-right). Table 3 summarizes the fit of these metrics (and four other metrics, see [54] for selected graphs) on: R2, RMSE (and RMSE%), and the number of conditions for which the error bars between model and human data overlap. Following [66], an ANOVA was applied to the model data to explore whether the same patterns of statistical effects were present in the data as observed in the human data. In this ANOVA, the model predictions for the best strategy alternative for each individual and each condition were treated as if generated by a participant. We applied a similar ANOVA structure as was used for the analysis of the empirical data—using a split mean analysis on typing speed to distinguish relatively fast typers from relatively slow typers. Table 4 reports these ANOVA results. In Table 3 we count what proportion of effects in the ANOVA of model data (Table 4) was similar to the ANOVA results of the empirical data (Table 2). In cases where one data set (i.e., model or human data) predicted a marginal effect and the other dataset predicted no effect or a significant effect, this was counted as explaining “half” of the effect. ANOVAs were not applied to the payoff score data, as the payoff function was an independent variable. An effect was counted as "wrong" in cases where the model predicted an effect that did not occur in the human results.

**Table 3:** Measures of fit between human performance and model predictions for optimal strategies.

	R2	RMSE	RMSE %	Nr error bars	ANOVA main effect correct	ANOVA main effect wrong	ANOVA inter-action correct	ANOVA inter-action wrong
Total time	0.89	5.2	44	4/8	3/3	1	3/3	2
Maximum deviation of cursor	0.90	11.11	13	2/8	4/4	0	1.5/2	1.5
Time outside of target	0.63	0.34	101	3/8	2.5/3.5	0	1.5/3.5	3
Maximum nr of digits typed	0.97	2.83	27	4/8	4/4	0	1/1.5	0
Typing visit time	0.91	0.56	21	3/8	3/3	0	0/1	0.5
Nr visits to tracking	0.94	4.58	164	0/8	4/4	0	1/1.5	2.5
Tracking time	0.90	0.79	63	0/8	3/3	1	0/0	0
Payoff score	0.76	5.3	93	2/8	NA	NA	NA	NA
**Mean**	0.86		66	2.3/8	96%	0.3 effects	64%	1.4 effects

See text for detail.

**Table 4:** Summary of statistical effects in model.

	Dependent variable						
	Total trial time	Maximum cursor deviation	Total time cursor outside target	Maximum nr of digits per visit	Mean visit time, typing window	Nr visits to tracking task	Mean visit time, tracking window
**Payoff function (P)**	***	***	***	***	***	***	***
**Noise (N)**	***	***	***	***	***	***	***
**Radius (R)**	***	***	***	***	***	***	***
**IKI group (I)**	***	***		***		***	***
**P x N**	***	***	***			***	
**P x R**	***		***			***	
**N x R**	***	.				***	
**P x I**				**	.		
**N x I**	**	.	.			.	
**R x I**	**	*				*	

Predictions are generated by treating model predictions for the best strategy alternatives as if they are generated by the corresponding participants (i.e., one datapoint per participant, per condition).

.: .05 < p < = .10;

*: .01 < p < .05;

**: .001 < p < .01;

***: p < = .001

Our analysis shows that on at least two metrics the human performance data was consistent with the performance predictions of the optimal model. First, R2 values were generally high (i.e., six out of nine measures were 0.89 or higher). Second, the ANOVA analysis of the model data produced similar main effects and interaction effects as the human data (e.g., 96% of main effects correct). Perhaps more importantly, the model predicts that on almost all the dependent variables there *should* be effects of payoff function, task characteristics (noise, radius), and individual differences in typing skill. Taken together, this analysis shows that the participants in the study were adopting strategies that were consistent with the predicted optimal performance model.

However, the model predictions of optimal performance did not always align perfectly with the human data. First, only in few conditions did the standardized error bars of the model and human data overlap (on average 2.3 out of 8), suggesting a difference between human and model data. Second, RMSE percentage scores were relatively high. Fig 5 helps in exploring where these differences occurred. For most measures, the largest discrepancy was in the hardest condition: high noise, small radius. Other discrepancies also occurred in the high noise, large radius condition. Inspection of the figures suggests that participants could have spent less time on the tracking task. This discrepancy might be attributed to the relative simplicity of the tracking model. For example, the model immediately started tracking when the tracking window opened, whereas participants might have needed some time to locate the cursor first. The model can be considered a model of idealized tracking performance, as it does not take these effects into account. More fine-grained data of human performance (e.g., eye-tracking data) is needed to model these effects. More detailed assumptions about tracking behavior would go beyond the level of granularity of the measurements in the current experiment.

### Exploratory analysis of learning to achieve optimum performance

**Fig 6:**
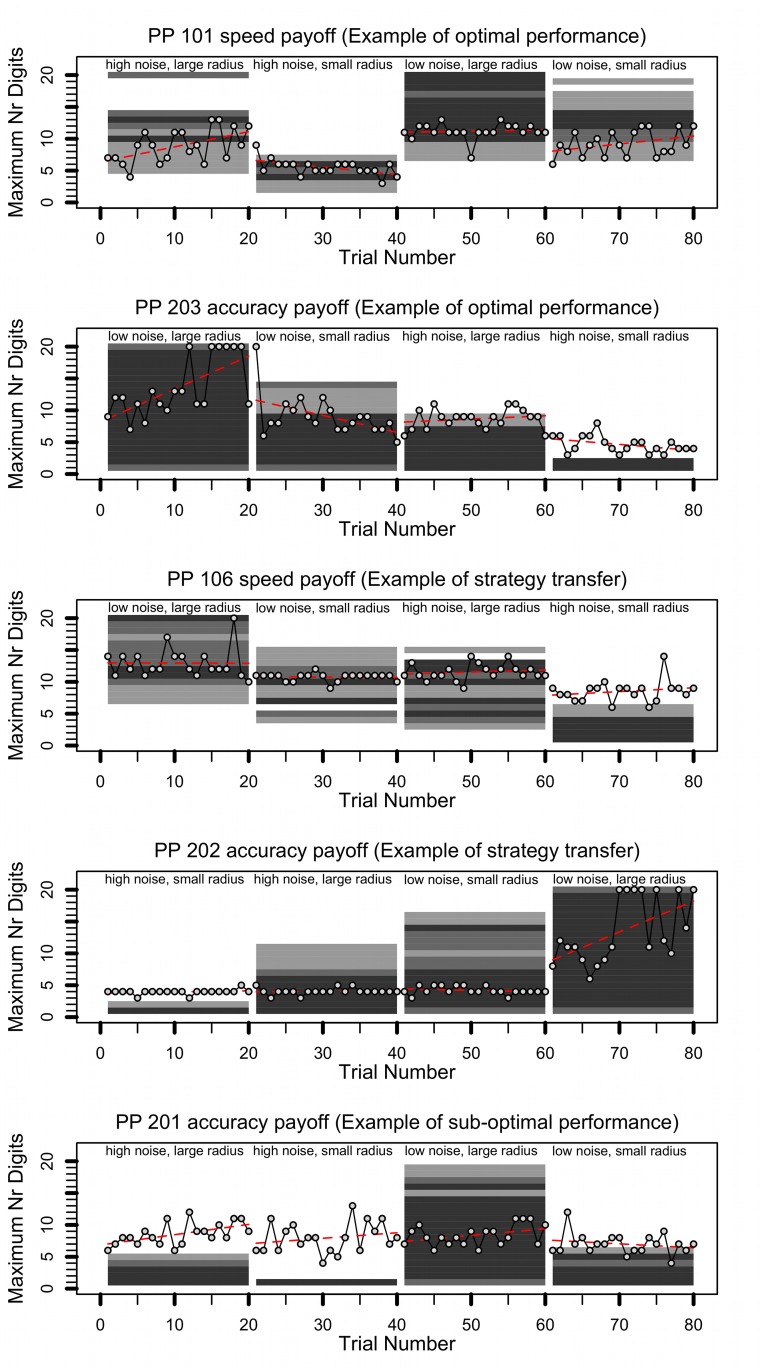
Progression of strategy choice over time versus model predictions of optimal choice. Data points show strategy per trial. Red lines provide fitted linear trend line. Dark rectangles highlight model predictions of optimal strategies, the darker the rectangle, the closer the strategy is to the optimal score (see text for details). Data is shown for five illustrative participants, see headings and text for description.

We also explored how the strategies that participants applied changed over time and how this relates to expected performance as predicted by the model. Fig 6 plots data for 6 representative participants, one participant per Figure (plots of all individuals can be found in Chapter 4 of [54]). The points plot the maximum number of digits per visit that was typed per trial over all trials (recall that the preceding analysis focused on performance during the last 5 trials of each condition; here we show data for all 20 trials of a condition). A red dashed line shows the trend line in the human data per condition, as predicted by a linear regression model.

Behind the data of the participant, rectangular areas show the model's prediction of relative success for each strategy in a particular condition. The three grey tones show strategies for which the best scoring strategy alternative (i.e., time spent on tracking) had a score that was maximum 0.5 pence (black), 0.1 pence (dark grey), or 0.2 pence (light grey) from the predicted maximum score for that specific condition and participant. Grey shade was always relative to a specific participant and a specific condition. Hence, a comparison of grey levels should be made within a participant and within a condition. Across conditions, different absolute scores might have been achieved.

If participants adopted optimal strategies, then their performance should lie inside the grey rectangular areas, especially inside the dark grey areas. However, the degree of overlap varied between participants and conditions. Some participants (e.g., participant 101 in the speed and participant 203 in the accuracy payoff condition, see Fig 6) adapted very well by almost always applying strategies that fell in the optimum region. Although these participants did not always apply optimal strategies on all trials, in general the trend lines suggest that over time they gradually reached optimal performance.

Some participants showed effects of strategy transfer between conditions. For example, participants 106 (speed payoff) and 202 (accuracy payoff) seemed to apply very similar strategies across conditions, which in general lead to good performance, but not necessarily optimal performance. Finally, some participants' strategy did not match predictions of the optimal strategy. For example, participant 201 consistently applied sub-optimal strategies on three blocks and did not vary strategies between conditions.

To quantify these results, we counted on how many trials the participants' chosen strategy fell in a grey area (i.e., where predicted score was less than 2 pence away from the optimum score) and applied an ANOVA analysis with payoff function, noise, and radius as factors. High scores were achieved on three times as many trials in the low noise condition (*M* = 15.04, *SD* = 3.64) compared to the high noise condition (*M* = 5.73, *SD* = 4.35), *F*(1, 22) = 121.57, *p* < .001, η_p_^2^ = 0.847. Performance was better when the radius was large (*M* = 14.04, *SD* = 3.36), compared to when it was small (*M* = 6.73, *SD* = 4.36), *F*(1, 22) = 73.07, *p* < .001, η_p_^2^ = 0.769. There was no effect of payoff function, *F* < 1. There was a significant interaction effect between payoff function and noise, *F*(1, 22) = 7.23, *p* = .013, η_p_^2^ = 0.247. There were no other significant interaction effects. Very similar effects were found when the analysis was performed when only counting strategies that achieved a score within 0.5 pence of the maximum strategy (i.e., that fall inside the dark black bars, see [54] for analysis).

These results suggest that how well participants performed in comparison with their own payoff curve (i.e., with the location of the maximum strategy) depended on the task characteristics, but not on the payoff function. When the tasks were relatively easy, due to low noise or a large radius, participants on average achieved a maximum score on more than half of the trials. The absence of a significant effect of payoff function in this analysis is good. It implies that the manipulation of payoff function did not pose any limitations on participants’ ability to adapt performance to the payoff function. Stated differently, if there were a significant effect of payoff function, it would suggest that participants applied more optimal strategies in one payoff condition compared to another payoff condition. This is not the case; participants were equally good in both payoff conditions.

**Fig 7:**
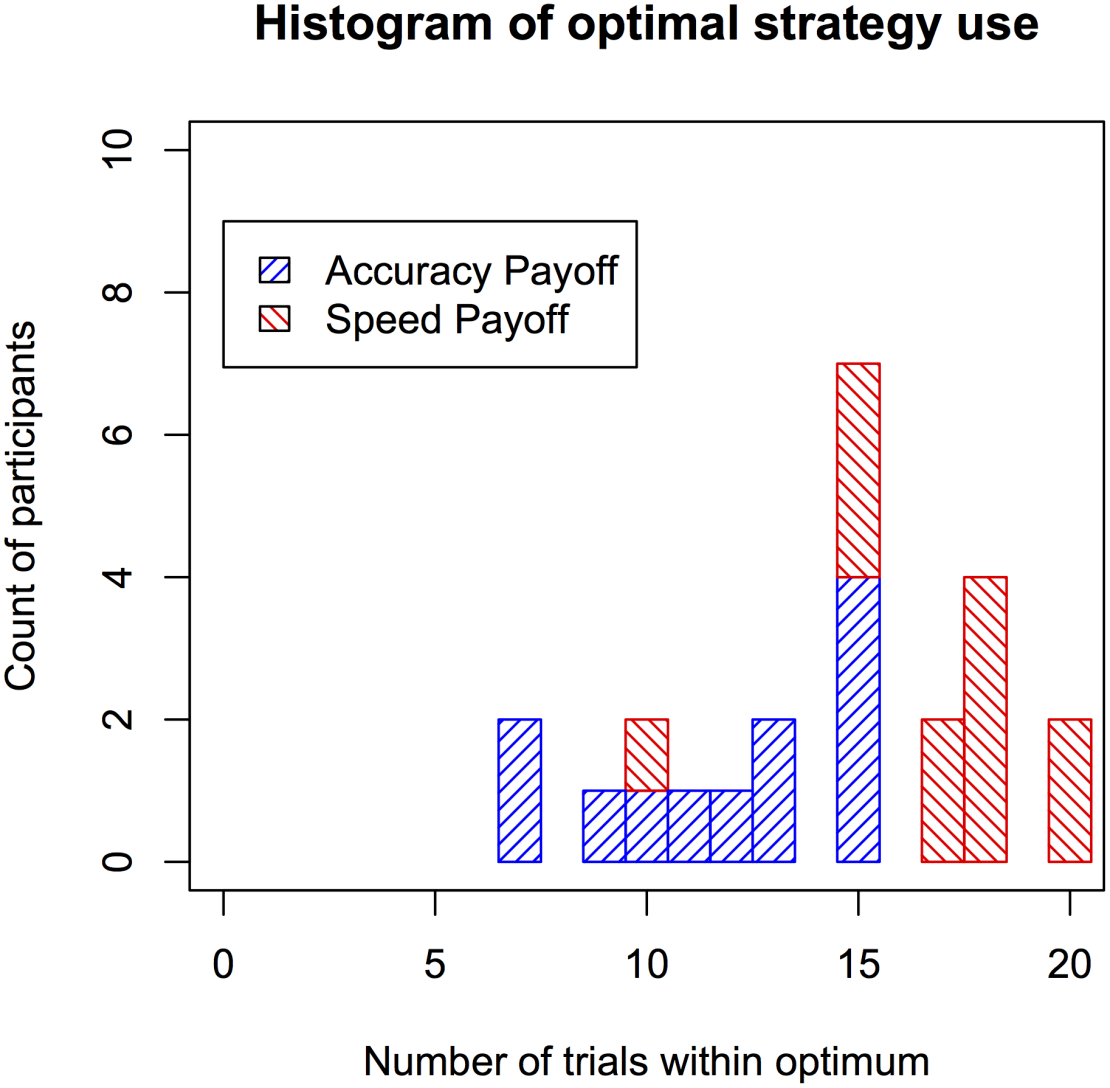
Histogram of how frequent participants applied the optimum strategy. Optimum strategies are those that achieved a score that was predicted to fall within 2 pence of the maximum score (i.e., that were highlighted in grey in Fig 6). Within each bar the proportion of participants from each payoff condition group is highlighted. For each participant only the last five trials of each condition are considered.

**Fig 8:**
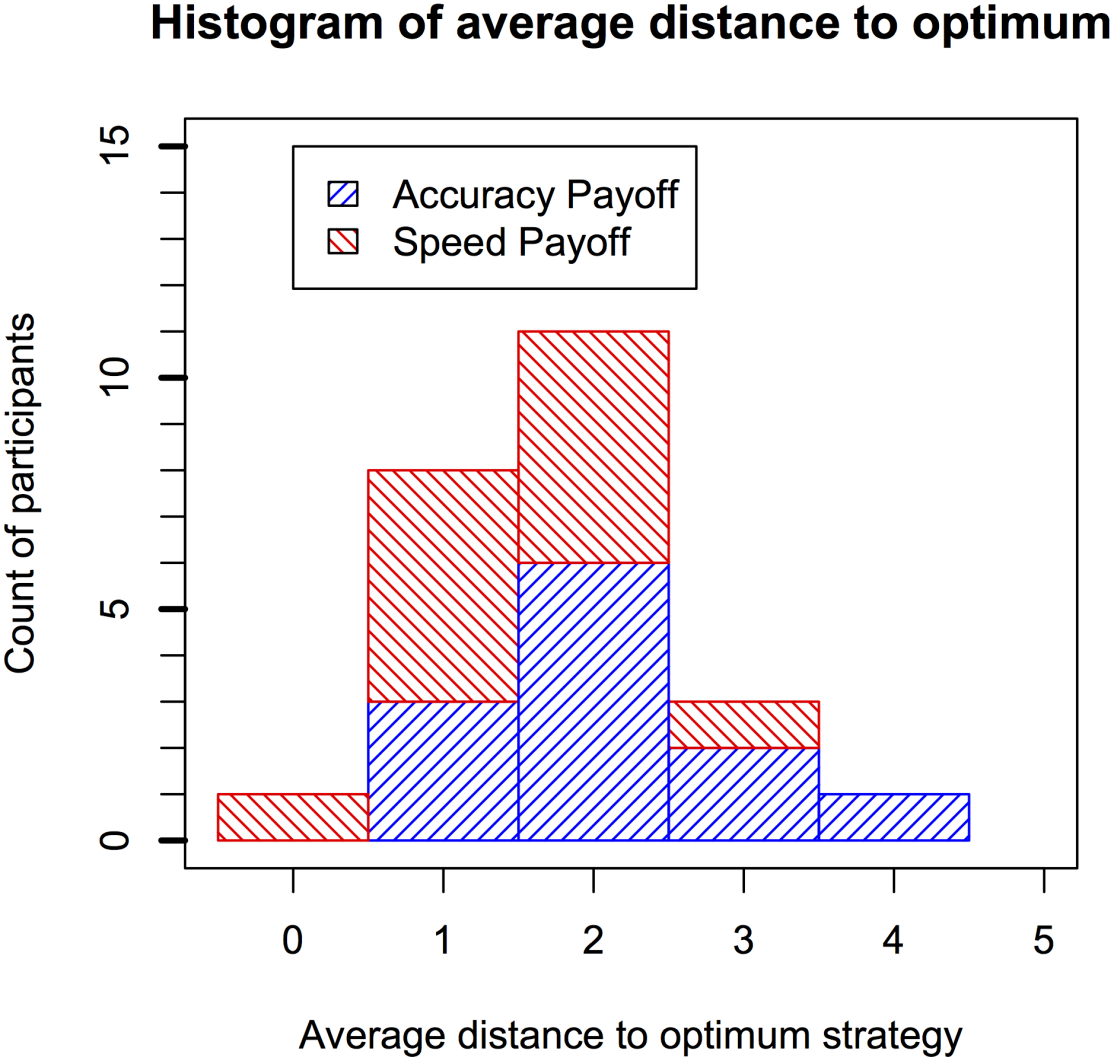
Histogram of average minimum distance from the predicted optimum strategies. Optimum strategies are those that were predicted to achieve a score that fell within 0.5 pence of the overall best strategy. Within each bar the proportion of participants from each payoff condition group is highlighted. For each participant only the last five trials of each condition are considered.

As a final analysis, we investigated whether there were individual differences in how frequently the optimum strategy was applied on the last five trials of each block (i.e., 20 trials in total). Optimum strategy was applied here as a strategy that fell in the grey zone of Fig 6 (i.e., with a predicted score within 2 pence of the predicted optimal score). The resulting histogram in Fig 7 suggest that in general, 21 out of the 24 participants applied an optimal strategy on at least half of the trials. Within each bar, the percentage of participants from each payoff condition is highlighted in a different color (accuracy: blue, right tilted lines; speed: red, left tilted lines). Participants in the speed payoff condition applied the optimal strategies more frequently. An analysis of the average minimum distance to the best strategy (i.e., the shortest distance between the applied strategy and the black bars in Fig 6) across participants is plotted as histogram in Fig 8. This data suggests that participants on average were only 2 digits away from a strategy that can be considered optimal given the constraints on performance.

## General discussion

### Summary of results

In an empirical study we demonstrated how dual-task interleaving performance is systematically influenced by task characteristics, monetary incentives, and individual differences in skill. People spend longer on tasks if this is needed either because of the task's difficulty (e.g., when the cursor moved fast), or when this matched their priorities as formalized through an incentive (e.g., when this task is more rewarding). They also calibrate their strategies to their own skill (e.g., typing speed).

Using a computational cognitive model we assessed how well participants chose strategies that were best suited for them given task characteristics, incentives, and individual typing skill. The model analysis suggested that participants adapted their performance in such a way as to achieve an (for them) optimum score, as evidenced by high correspondence between the trend in the model and human data (e.g., high R^2^ and correspondence in ANOVA results). However, the exact strategies that participants applied were not yet the ones that, on average, achieved the highest mean score, as evident in for example relatively high RMSE values.

An analysis of the learning path gave three explanations for why performance did not always achieve the best scores. First, participants sometimes were still adapting their performance to the task at hand by the end of the block. Second, some participants transferred strategies from one block to the next and hardly adapted it to the circumstances. For some participants this was because these strategies optimized, or at least satisficed [67], performance (e.g., see performance of participants 106 and 202 in Fig 6), for others there was no clear explanation for why these strategies were applied. Third, the number of times that a participant applied the optimal strategy was influenced by the task characteristics. On harder tasks (e.g., small radius, high cursor speed), participants were relatively less successful in achieving the optimum score.

### Relationship to existing literature

Systematic influence of task characteristics, in particular task difficulty (e.g., [21,26–28]), on dual-task performance has been well-documented. Consistent with this work, we show how task characteristics influences performance in our set-up: performance declines when tasks are more demanding. In addition, task characteristics influence the strategies that participants choose to interleave between tasks. More time is spent on the more challenging tasks.

Incentives were used here to formalize participants' objective (cf. [18]) and to assess in an objective way whether participants achieved the best scores they could. This provides support for the notion that rational agents optimize their performance so as to maximize their payoff [18,35,53–56,68]. We showed that incentives have consequences for the strategies that are selected for interleaving attention and for performance on each of the individual task (e.g., total time spent typing, and maximum deviation of a cursor). Although participants adapted their performance towards optimal performance, they did not reach the overall optimum strategy in all cases. The computational models allowed us to identify reasons why this happened: strategy transfer and longer learning times.

We also found that individual differences in skill influenced performance, building on recent observations to include these in our understanding of multitasking (e.g., [10,11,41,52]). Our modeling work is among the first efforts to demonstrate how individual skills systematically influence the strategies with which tasks are interleaved, and thereby performance [41,52,54].

It can sometimes be hard to determine "task difficulty" independently from "skill". For example, cooking a steak exactly medium rare is easy for a seasoned chef, but might pose a significant challenge for a novel cook. In the later case we would perhaps call the preparation of a steak a "difficult" task, relative to the (lower) skill level of the novel cook. In general, experience and training can help to develop skills and can turn a difficult task into a simpler one. Various studies have looked at how the acquisition of new skills can impact performance in dual-task settings (e.g., for recent examples see [69,70]).

In our experiment, skill and task difficulty can more easily be distinguished in an objective manner. We manipulate inherent properties of the tracking task, that make the task relatively more easy (e.g., low noise, large radius conditions) or relatively more hard (e.g., high noise, small radius conditions). For the typing task, we do not manipulate the difficulty (e.g., no strings are harder than others). However, we observe that there are differences in typing skill: some participants type faster than others. As typing is a skill that is acquired over years of practice we did not expect that there is significant typing skill acquisition during our experiment (cf. e.g., [59,60]).

### Limitations and future work

The modeling analysis suggested that participants did not consistently apply strategies that the model predicted to be optimal for them given the constraints on performance. If we assume that the model is correct, this discrepancy might be due to several shortcomings in the experiment. First, some participants needed more trials to learn the optimal strategy. Providing more trials for learning would specifically be successful if during some of these trials participants had time to freely explore the value of different strategies without being penalized for this. This can for example be done by using a no-choice/choice paradigm (e.g., [71–74]) in which the participant is first forced to apply specific strategies (no-choice) to explore performance of various specific strategies, and then allowed to choose their own strategies (choice), given their knowledge of likely success-rate.

Performance feedback was only given at the end of the trial. More feedback might be needed to guide the learning of new strategies. Providing feedback during trials (instead of only at the end) increases the amount of information that is available, as in [32]. Such feedback is particularly useful in the high noise condition, where more variability in the position of the cursor makes the outcome of specific strategies more variable from one trial to the next.

More generally, the timing, objective function, and magnitude of rewards can influence a model's predictions of optimal behavior [33] and influence whether participants can find the optimum (as for example studied in the context of melioration and maximization of performance, see e.g., [75,76]). Stated differently, different performance might occur when rewards are only a couple of cents (as in our study) versus hundreds of dollars (i.e., a difference in magnitude). To reduce ambiguity for the participant and the modeler on what should be optimized, we provided explicit numeric feedback, so as to have a "golden standard" (cf. [18,32,52–56]).

One conclusion from our analysis is that human participants do not always seem to perform optimally. However, it might also be that human performance was optimal, but that our model was not accurate. For example, although we assumed that participants optimized the objective payoff function, perhaps internally other factors (e.g., motivation, interest) were optimized. Following this line of reasoning, our model can be seen as a method of capturing important aspects of the task environment, individual differences, and the payoff and providing a detailed, *normative* assessment of what should constitute "rationally bounded behavior" given these constraints. The deviations of the optimal predictions are interesting, as they pose new questions for study of human multitasking behavior.

The above consideration reflects a broader concern within the cognitive science community of identifying the appropriate normative theory (or using Marr’s parlance: computational level of explanation [64]). Take for example the classic problem of the Wason selection task [77,78]. In this task, participants need to turn around a set of cards to test a logical rule that is provided by the experimenter (e.g., "All cards that have a vowel on one side, have an even number on the other side"). A consistent finding is that participants do not follow the rules of logic in this task. Although this could be interpreted as a deviation from rational behavior, later analyses using a different model and theory demonstrated that behavior in the selection task can actually be cast as *optimal* data sampling behavior ([79,80], for a more recent version see [56]). That is, this work demonstrated that behavior that was initially believed to show (and was modeled as) a deviation from optimality could in fact be seen as optimal. In a similar vein, rational explanations have recently been developed for other tasks were the assumption has been that people act suboptimally (e.g., the gambler's fallacy [81] and anchoring [82]).

It is possible that behavior in our task is also more frequently optimal when judged on a different criterion than what was used in our analysis. To avoid strong assumptions on human behavior, the components of the model were grounded in measurements that were taken in single-task (e.g., for interkeypress intervals) or that were specified in preceding models of this task setting in which a different payoff function was used (e.g., parameters for the control of the joystick and for switch costs [18]). In this way, we attempted to craft a model that did not go beyond the empirical data.

That said, more detailed insights might be gained when the model is refined further. Depending on the nature of the revision, alternative predictions regarding optimality might arise. We see four general ways in which the model can be refined. First, more details of the underlying psychological processes and the moment-to-moment performance could be given for most components of the model. Such theories can provide an account of performance at different levels of abstraction [62]. For example, Zhang and Hornof [41] have developed models that predict performance of various 'microstrategies' for dual-tasking (i.e., systematic combinations of cognitive processes at the millisecond to second level [83]). Similarly, our model does not incorporate a theory of effort or motivation. It provided a normative account for what performance might look like for different strategies for interleaving between tasks. It did not account for different effort levels that can be applied, given the choice for a specific strategy. It is possible that participants adhered to general principles such as a minimization of effort [84,85] and a richer model, with more assumptions, is needed to account for this.

Second, the model could be calibrated to take more variability of performance into account. For example, most of the model's parameters are set to a mean value (e.g., mean typing speed). This can be changed to take trial-to-trial variability into account (e.g., by sampling values from a distribution).

Third, the strategy space might be broadened in two ways. First, the model was only used to explore simple strategies in which a consistent number of digits was typed during each visit. However, participants might have used more complicated strategies. For example, they might have varied the number of digits they typed per visit, or they might have changed the number of digits they typed based on the occurrence of "structure" in the number (e.g., see [24] for an example where task structure influences interleaving). More fine-grained measurements (e.g., eye-tracking) are needed to accurately model such strategies. As the current model explored performance of extreme strategies (e.g., no interleaving, and interleaving after every digit), as well as many strategies in between these extremes, it is expected that performance of more "complex" strategies falls in the same range as the current model predicted (cf. the bracketing approach see [43,86]).

Fourth, the model could be improved by incorporating a formal theory of how people learn to adapt to constraints over time. Although some theories of learning in multitasking have been proposed (e.g., [58,87]), these theories are not yet at a level of sophistication such that they can directly be applied to the current context. In particular, it is unclear at what level of granularity feedback on performance is cognitively processed, and how experience with one strategy is generalized to other strategies. Insights from hierarchical reinforcement learning might prove valuable here, as such models learn both the utility of small consistent action units, while at the same time learning the utility of larger units (e.g., strategies) that are formed out of these smaller units [88].

## Conclusion

We provided a detailed analysis of how people adapt their interleaving strategies in a dual-task setting to three factors: task characteristics (noise, radius), individual differences in skill (e.g., typing speed), and incentives (a formal way of capturing objective or priority). The modeling analysis suggests that people adapt their performance in such a way as to try and maximize the payoff value. This is not to say that performance was optimal on every trial. Several explanations have been given for this. Some are related to the learning process (e.g., strategy transfer and exploitation of successful strategies), others might have to do with the difficulty of the task (e.g., the noise in the feedback).

## Supporting Information

S1 File Script and data for analysis of the empirical data. The zip-file contains a R script and .Rdata file that can be used to analyze the empirical data. The script explains the structure of the data file.
(ZIP)
